# Are Individuals with Low Trait Anxiety Better Suited to On-Call Work?

**DOI:** 10.3390/clockssleep2040035

**Published:** 2020-11-12

**Authors:** Madeline Sprajcer, Sarah M Jay, Grace E Vincent, Xuan Zhou, Andrew Vakulin, Leon Lack, Sally A Ferguson

**Affiliations:** 1 Appleton Institute, School of Health Medical and Applied Sciences, Central Queensland University, Wayville 5034, SA, Australia; sarahmaryjay@gmail.com (S.M.J.); g.vincent@cqu.edu.au (G.E.V.); sally.ferguson@cqu.edu.au (S.A.F.); 2 Australian Centre for Precision Health, University of South Australia, Adelaide 5000, SA, Australia; xuan.zhou@unisa.edu.au; 3 Adelaide Institute for Sleep Health, College of Medicine and Public Health, Flinders University, Adelaide 5042, SA, Australia; andrew.vakulin@flinders.edu.au; 4 Woolcock Institute of Medical Research, Centre for Sleep and Chronobiology, Sydney 2037, NSW, Australia; 5 Adelaide Institute for Sleep Health, College of Education, Psychology, and Social Work, Flinders University, Adelaide 5042, SA, Australia; leon.lack@flinders.edu.au

**Keywords:** anxiety, on-call, qEEG, stress

## Abstract

Research has indicated that individuals with certain traits may be better suited to shiftwork and non-standard working arrangements. However, no research has investigated how individual differences impact on-call outcomes. As such, this study investigated the impact of trait anxiety on sleep and performance outcomes on-call. Seventy male participants (20–35 years) completed an adaptation night, a control night, and two on-call nights in a laboratory. Trait anxiety was determined using the State Trait Anxiety Inventory (STAI) X-2, and participants completed the STAI X-1 prior to bed each night to assess state anxiety. Sleep was measured using polysomnography and quantitative electroencephalographic analysis. Performance was assessed using a 10-min psychomotor vigilance task (PVT) performed each day at 0930, 1200, 1430 and 1700 h. Data pooled from three separate but inter-related studies was used for these analyses. Results indicated that the effects of trait anxiety on state anxiety, sleep and performance outcomes on-call were generally limited. These findings suggest that on-call outcomes are not negatively affected by higher levels of trait anxiety.

## 1. Introduction

Shift work and non-standard working arrangements have become ubiquitous in modern workplaces, and as such have been an increasing focus of sleep and circadian research. This growing body of research demonstrates a range of potential negative consequences of non-standard working arrangements, including shortened or disturbed sleep, poorer cognitive performance, and overall ill health [1]. With this increased understanding of negative outcomes comes the opportunity to understand protective factors or individual differences that may reduce susceptibility to these adverse consequences.

On-call is one non-standard working arrangement, often employed by workplaces to minimize costs while ensuring continuous coverage [2,3,4]. On-call periods often occur overnight, when workloads may be reduced and/or variable. Overnight on-call periods may result in poor or shortened sleep, often as a result of disturbed sleep opportunity [2]. Even in the absence of calls, the potential for disruption (i.e., receiving a call) has been linked with increased anxiety and apprehension in on-call workers [5]. This has been seen, for example, in subjective reports from firefighters [6,7], and on-call rail workers [8]. Anxiety and apprehension have been linked with poor sleep [9], and may explain poor sleep outcomes seen when on-call in the absence of overnight calls [10].

Increased anxiety and poor sleep associated with on-call work may have significant negative effects on the productivity, performance, safety, and mental health of employees [5,11,12]. It is therefore critically important to understand any individual factors that are associated with an improved capacity to cope with on-call work. Coping, or tolerance, may be reflected by fewer decrements to sleep, performance, or psychological factors (e.g., anxiety). Research into shift work tolerance, including the ability to manage factors such as night work, shortened sleep, and roster types [13] has shown that individuals who are younger, and who have lower scores on neuroticism and trait anxiety scales, are more likely to be able to tolerate non-standard working hours [13]. However, there is no research specifically addressing traits that may be linked with on-call outcomes (e.g., sleep, performance, or anxiety).

One key factor that may be associated with the capacity to cope with on-call work is trait anxiety. Trait anxiety refers to an individual’s inherent, or baseline level of anxiety [14], which reflects both an increased vulnerability to psychopathology, and altered neurobiological factors [15]. The potential for trait anxiety to impact on-call tolerance reflects the current understanding of shift work tolerance [13,16], in addition to research outside of non-standard working arrangements. Higher trait anxiety is associated with a reduced ability to manage stressful situations [17], including work stressors [18] and even large-scale natural disasters [19]. In addition, individuals with higher trait anxiety may be more reactive to stressful situations and, as such, when faced with a stressor, may have a heightened state anxiety response [17,20,21]. Indeed, Stokes and Kite [18] note that “the trait anxious person is more likely to perceive a given situation as threatening, and to react to this apparent threat with higher levels of state anxiety” (p. 25). As such, higher levels of trait anxiety may be associated with greater state anxiety during on-call periods. Not only may this suggest poorer psychological outcomes, but it may result in poorer or shortened sleep, and subsequent decrements to both cognitive and work performance [22].

In addition to the potential effects of trait anxiety on individual outcomes on-call, there are factors inherent to on-call work that may affect this relationship. These include perceptions of the likelihood of receiving a call overnight [23], the nature of the task that the individual will be required to perform upon waking [24], and concern about missing the alarm for the on-call incident [25]. The aforementioned factors have been shown to affect anxiety, sleep, or performance outcomes during on-call work. For the current study, we analyzed data from the three interrelated studies to understand how trait anxiety may affect outcomes under different on-call conditions. By examining individual factors (i.e., trait anxiety) using statistical analyses that differ from previous published works, we have addressed a novel research question—whether individuals with lower trait anxiety are better suited to on-call work. Each study was analyzed separately to understand the impact of the specific on-call conditions.

This study aimed to investigate if individuals who have higher trait anxiety are more susceptible to the negative effects of being on-call, and how the on-call context may affect this relationship. Specifically, we hypothesized that higher trait anxiety would be associated with higher pre-bed state anxiety during on-call periods, in addition to being associated with poorer sleep and poorer next-day cognitive performance. Furthermore, we anticipated that this relationship would exist under a variety of on-call conditions.

## 2. Materials and Methods

### 2.1. Participants

Participants were 70 healthy males aged between 20 and 35 years (*n* = 24 (Study 1); *n* = 22 (Study 2); *n* = 24 (Study 3)). Participants were screened to ensure that they were non-smokers, had a BMI of under 30 kg/m^2^, and did not have any medical concerns likely to impact sleep. Participants were excluded if they had high daytime sleepiness (Epworth Sleepiness Scale score > 10) [26] or showed signs of sleep disturbance via a Pittsburgh Sleep Quality Index score of >5 [27]. Participants were also screened with the Depression Anxiety Stress Scale [28] and the Morningness Eveningness Questionnaire [29] to exclude individuals who were outside of standard ranges on either scale. Participants were excluded if they had Depression Anxiety Stress Scale scores over 20 on the depression subscale, over 14 on the anxiety subscale, or 25 on the stress subscale—scores over which indicate ‘severe’ or ‘very severe’ outcomes. Participants were also excluded if they were classified as being a ‘definite morning type’ (score 70–86) or a ‘definite evening type’ (score 16–30) on the Morningness Eveningness Questionnaire, as these scores reflect extreme chronotypes. Data collection for Study 1 occurred from January–June 2016, Study 2 from July–December 2016, and Study 3 January–June 2017. Participants were allocated to either Study 1, 2, or 3 based on their availability during scheduled study times. All participants in all studies were required to meet the same eligibility criteria as described above.

### 2.2. Protocol

The data reported in this paper come from three independent but related, four-night studies that were conducted in the Appleton Institute sleep laboratory in Adelaide, Australia (2016–2017). Ethical approval was obtained from the CQUniversity human research ethics committee (H15/08–158).

Some data from these studies have been reported elsewhere [23,24,25]. However, the present analyses aim to answer a novel research question (i.e., the relationship between trait anxiety and on-call outcomes) based on previously unreported trait anxiety data.

**Figure 1:**
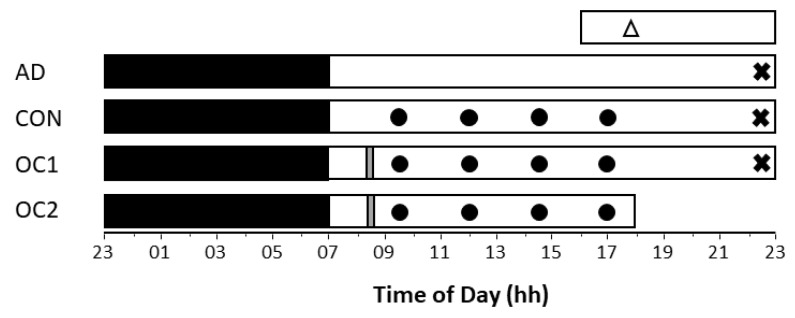
Protocol diagrams of Study 1, 2, and 3. AD = Adaptation night, CON = Control night, OC1 = On-call night 1, OC2 = On-call night 2. 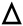 = Trait anxiety questionnaire (State Trait Anxiety Inventory (STAI) X-2). 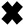 = State anxiety questionnaire (STAI X-1) and on-call instructions administered. 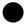 = psychomotor vigilance task (PVT). 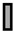 = High or low stress task performance (Study 2 only). 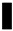 = Sleep opportunity.

All participants completed an adaptation, a control and two consecutive on-call nights (*n* = 24 for each study). Bedtime on all nights was 2300 h, with a 0700-h wake time (see Figure 1). As this was a time-isolated facility, participants were unaware of the time at which they were woken each morning. On control nights, participants were told that they were not on-call and would be having a full night of sleep. On on-call nights, participants were told 45 min prior to bedtime that they were on-call, and what on-call condition they were in. Despite being on-call on relevant nights, participants were only ever ‘called’ at the end of their eight-hour sleep opportunity.

In all studies, the two on-call nights were counterbalanced, but different on-call conditions were included in each of the three studies. Pre-bed instructions were the only difference between each study.

- In Study 1, participants were told before bed on one on-call night that they would definitely be called during the night, and on the other that they may be called (likelihood of being called; conditions: definitely, maybe).
- In Study 2, on one on-call morning, the participant was required to do a 5-min silent reading task (a low stress task), and on the other, they were required to do a 5-min speech in front of a researcher (a high stress task). Participants were told about these activities before bed on the preceding night (task stress; conditions: high stress, low stress).
- In Study 3, participants were demonstrated a loud (105 dB(A)) alarm and a quiet (white noise) alarm prior to the study. Researchers told them that no one had ever missed the loud alarm, but that some previous participants had missed the quiet alarm. On one on-call night, participants were told they would be woken by the loud alarm, and on the other, the quiet alarm. This study investigated the effects of the perceived chance of missing the on-call alarm (chance of missing the alarm; conditions: high chance, low chance).

### 2.3. Measures

#### 2.3.1. Anxiety

Trait anxiety was measured shortly following first arrival at the laboratory by the State Trait Anxiety Inventory form X-2 [30]. This questionnaire requires respondents to answer questions relating to how they feel generally, including items such as “I feel pleasant” or “I wish I could be as happy as others seem to be”. Positive items were reverse-coded for scoring, according to standard practice. State anxiety was measured prior to bed on control and on-call nights by the State Trait Anxiety Inventory form X-1 [30]. This questionnaire asks similar questions to the X-2 form, but requires participants to respond in terms of how they feel right now. Scores on both questionnaires range from 20–80, with higher scores reflective of higher trait or state anxiety. Anxiety of clinical significance is reflected by scores over 39 [31].

#### 2.3.2. Sleep

Sleep was measured using polysomnography. Electrodes were applied to the head, face, and torso in a standard montage to take electroencephalographic (EEG), electromyographic (EMG) and electro-oculographic (EOG) recordings. The channels used were C3/M2, F4/M1 and O2/M1, with Cz also recorded for use in a quantitative EEG analysis. Standard criteria were used for scoring each 30 s epoch [32] and a trained technician who was blinded to study conditions performed all scoring. Variables included total sleep time (TST), sleep efficiency (SE, time asleep as a proportion of the total sleep opportunity), wake after sleep onset (WASO), sleep onset latency (SL), latency to 10 min of sleep, REM (rapid eye movement sleep) latency and latency to N3. The proportion (as a percentage of total sleep time) and minutes of each sleep stage (N1, N2, N3, REM, NREM (non-REM)) were also calculated.

#### 2.3.3. Quantitative EEG Analysis

Quantitative EEG analyses were performed on the polysomnographic output of the Cz electrode for each night. This involves use of a validated algorithm [33] and fast Fourier transformations (FFT) to assess the frequency composition of each sleep stage. Artefacts were automatically removed from the polysomnographic output, with manual checking performed for 10% of the recordings. Manual checking revealed an accuracy of 98.0%. Frequency bands included delta (0.5–4.5 Hz), theta (4.5–8.0 Hz), alpha (8.0–12.0 Hz), sigma (12.0–15.0 Hz), and beta (15.0–32.0 Hz) [34]. Due to its greater sensitivity to smaller changes in sleep than traditional polysomnography, a quantitative EEG assessment was used to reveal the makeup of each sleep stage in terms of frequency composition. A sleep stage with a lower frequency makeup (e.g., a higher proportion of delta) may be indicative of deeper sleep, while a higher proportion of beta may be indicative of lighter, less restful sleep [35]. The variables included in these analyses were a delta/alpha ratio, with higher scores indicative of increased slowing, and an overall EEG slowing ratio variable, calculated by ((delta + theta)/(alpha + sigma + beta)). These ratio variables were calculated for both REM and NREM sleep.

#### 2.3.4. Performance

Cognitive performance was assessed using a 10-min psychomotor vigilance task (PVT) [36], administered at four time points on the control and both on-call days (0930, 1200, 1430, 1700). PVTs lasted for 10 min and are a measure of sustained attention and vigilance that is sensitive to sleep loss [36]. Variables include mean reciprocal reaction time (RRT, reaction speed), mean fastest 10% of reaction time, mean slowest 10% of reciprocal reaction time, and lapses (>500 ms) [37]. Reciprocal reaction time is 1/reaction time in milliseconds and is a standard outcome variable of the PVT. Learning effects were accounted for by three practice sessions on the adaptation day [38].

### 2.4. Data Analysis

All statistical analyses were performed using R, and all models were fitted using the LmerTest package (version 3.0) [39]. Each study was analyzed separately.

Mean trait and state anxiety for each study and condition were assessed via one-way ANOVA and post hoc Bonferroni analyses. To investigate the relationship between trait anxiety and on-call outcomes (state anxiety, sleep, and cognitive performance), we fitted separate linear mixed-effect models. These models compared the correlations between trait anxiety and outcome variables. Fixed effects included condition (3 levels: control, on-call condition 1, on-call condition 2), trait (as a covariate) and condition × trait interaction, and a random effect of participant. To account for the covariance between repeated measures within the same participant, the random effect imposed a compound symmetry variance-covariance structure to the data. By fitting these models, we estimated the effect of trait anxiety on the outcome measures under all three conditions of a given study.

For each outcome variable, we tested whether the effect of trait anxiety under the control condition of a study was different from zero (a slope of zero indicating no relationship between trait anxiety and the given outcome variable); and whether the effects of trait anxiety under the two on-call conditions deviated from the relationship between trait anxiety and the outcome variable in the control condition. Each contrast involved a t-test, the degree of freedom of which was approximated using the Satterthwaite method. For all tests, the alpha level was set at 0.05.

In plain terms, these analyses found the slope of the relationship between trait anxiety and an outcome variable. For control conditions, this slope was compared with zero. In the on-call conditions, this slope was compared to the control slope of the same study (acting as a baseline).

## 3. Results

### 3.1. Demographics

Participant characteristics can be seen in Table 1.

### 3.2. Mean Trait and State Anxiety

No significant differences were found between studies in trait scores on the State Trait Anxiety Inventory (STAI) form X-2 (mean ± SD) (Study 1: 30.8 ± 4.3, Study 2: 30.5 ± 5.5, Study 3: 32.2 ± 4.6), F (2, 143) = 1.63, *p* = 0.200.

Significant differences were found between studies for overall levels of state anxiety, F(2, 212) = 45.44 *p* < 0.000. Bonferroni post hoc testing revealed that participants in Study 2 (which investigated task stress) had higher overall pre-bed state anxiety (40.8 ± 5.0) compared with Study 1 (which examined call likelihood) (33.6 ± 6.5), *p* < 0.000 and Study 3 (which investigated the chance of missing the call) (32.4 ± 5.5), *p* < 0.000. State anxiety in the control condition in Study 2 (41.3 ± 5.5) was found to be significantly different from the control conditions for Study 1 (32.0 ± 5.7), *p* > 0.000 and Study 3 (29.4 ± 4.1), *p* < 0.000.

### 3.3. Trait Anxiety and State Anxiety

**Figure 2:**
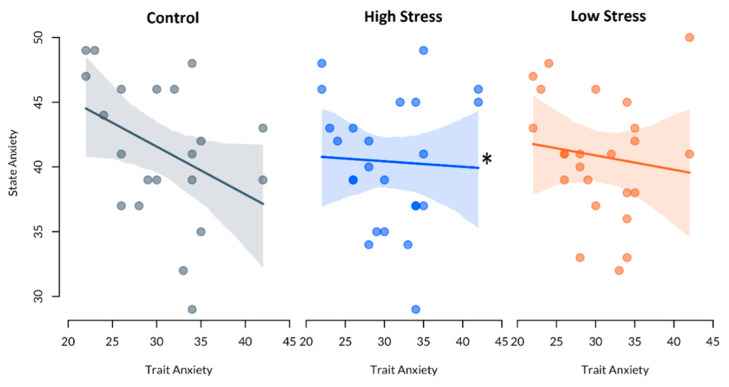
Relationship between state anxiety and trait anxiety in Study 2 conditions. * Significantly different from control relationship. Note R^2^ < 0.1.

Linear mixed-effects models showed that there was no significant effect of trait anxiety on state anxiety in Study 1, or Study 3, *p* > 0.05. In Study 2, there was a significant effect of trait anxiety on state anxiety in the high stress condition, t(41.39) = 2.43, *p* = 0.019, compared with control. However, the effect of trait anxiety on state anxiety in the control condition of this study was not significant, t(31.74) = −1.95, *p* = 0.060, nor was this relationship significant in the low stress condition when compared with control, t(41.39) = 1.93, *p* = 0.061. See Table 2 and Figure 2.

### 3.4. Trait Anxiety and Sleep Outcomes

**Figure 3:**
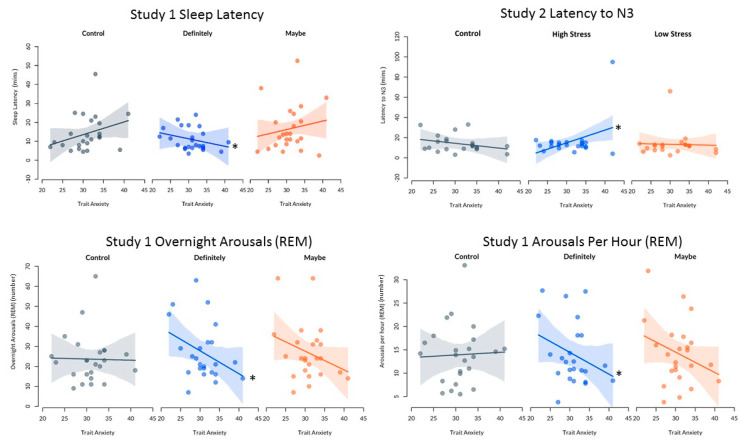
Statistically significant relationships between trait anxiety and sleep variables. * Significantly different from control slope. Note R^2^ < 0.1.

Linear mixed effects models showed that the impact of trait anxiety on sleep was significantly different under control as compared with on-call conditions for several key sleep outcome variables. These significant differences were seen for sleep latency, latency to N3, number of arousals during REM overall, and number of arousals per hour during REM. See Figure 3 and Table 3. However, it must be noted that when a sensitivity analyses was performed and outliers removed, there was no longer a statistically significant effect of condition on latency to N3 in Study 2, *p* > 0.05. This suggests that the impact of condition on latency to N3 is not robust.

No significant differences between control and on-call conditions were seen for TST, SE, WASO, latency to REM, latency to 10 min of sleep, minutes and proportion of each sleep stage (N1, N2, N3, REM, NREM), arousals during sleep and NREM, awakenings and stage shifts, *p* > 0.05. Similarly, no significant differences in the impact of trait anxiety on sleep between control and on-call conditions were found for quantitative EEG ratio outcomes, *p* > 0.05.

### 3.5. Trait Anxiety and Cognitive Performance

A significant relationship was found between trait anxiety and mean RRT in the control condition of Study 3, β = −0.04, t(38.42) = −2.07, *p* = 0.045 (i.e., higher trait anxiety was associated with slower reaction times). In comparison with this relationship, no significance was seen in the low, β = −0.02, t(44.00) = 1.12, *p* = 0.270, or high chance of missing the alarm conditions, β = −0.00, t(44.00) = 1.04, *p* = 0.306. No significant differences in the relationship between trait anxiety and mean reciprocal reaction time were found between control and on-call conditions in Study 1 or 2, *p* > 0.05.

In Study 3, significant deviation from zero in the slope of the relationship between trait anxiety and lapses was seen in the control condition, β = 0.17, t(35.04) = 2.67, *p* = 0.012. (i.e., higher anxiety was associated with more lapses). Compared with this relationship, there was a significantly smaller positive relationship between trait anxiety and lapses in the low chance of missing the alarm condition, β = 0.06, t(44.00) = −2.16, *p* = 0.036. However, there was no significant difference between the control slope and the high chance of missing the alarm condition, β = −0.02, t(44.00) = −1.40, *p* = 0.167. Additionally, when analyses were performed comparing lapses between conditions in Study 3 excluding the visible outlier, no statistical significance was seen, *p* > 0.05. Additionally, no significant differences in the relationship between trait anxiety and lapses on the PVT were seen in Studies 1 or 2.

A significant difference was seen in the relationship between trait anxiety and the mean slowest 10% of reciprocal reaction time compared with zero in the control condition of Study 3, β = −0.06, t(47.75) = −2.39, *p* = 0.021. This relationship was not significantly different from control in either the low, β = −0.01, t(44.00) = 1.71, *p* = 0.094, or high chance of missing the alarm condition, β = 0.01, t(44.00) = 0.99, *p* = 0.328. No significant differences in the relationship between trait anxiety and lapses on the PVT were seen in studies 1 or 2.

**Figure 4:**
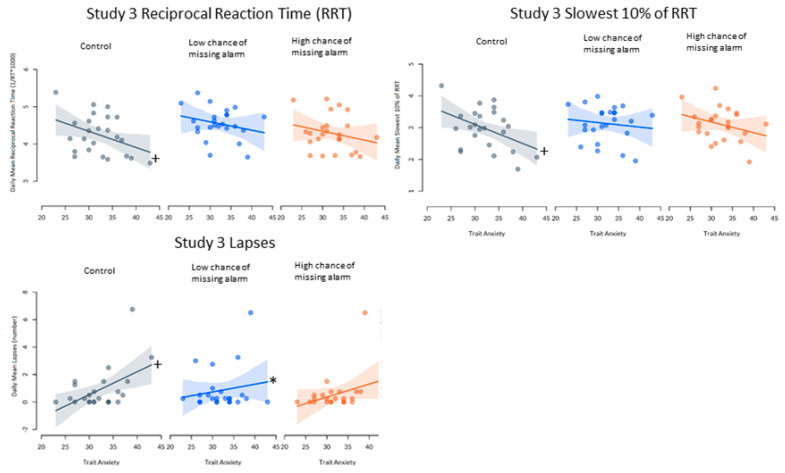
Relationships between trait anxiety and performance variables in Study 1, 2 and 3. + Significantly different from slope of zero. * Significantly different from control slope. Note R^2^ < 0.1.

No significant differences in the relationship between trait anxiety and the mean fastest 10% of reaction time on the PVT were found between conditions in Study 1, 2 or 3 (See Figure 4).

## 4. Discussion

The aim of these analyses was to understand if individuals who have lower trait anxiety are better able to cope with on-call working arrangements. This is important to understand due to the potentially detrimental impacts of on-call work on worker performance, productivity, safety, and health. Results indicated that trait anxiety was the same across all three studies, suggesting that all individuals were starting from a similar baseline level of anxiety. As all participants were screened for severe levels of anxiety prior to participation, this result is not surprising. However, the state anxiety experienced by participants across all conditions in Study 2 was significantly higher than in either Study 1 or Study 3. Had these increases been limited to Study 2’s on-call nights (high and low stress conditions), we may have posited that being required to perform a specific task upon waking while on-call may be responsible for increased pre-bed state anxiety. However, as the level of state anxiety experienced prior to bed in the control condition was similarly heightened compared with trait anxiety levels, we cannot surmise that it was the on-call conditions that resulted in significantly higher state anxiety. Additionally, participants were only informed prior to bed on the on-call nights that they would be required to perform the speech task, and as such were unaware of this requirement on the control night.

Demographic composition, including age, BMI and nationality, were equally distributed between studies, suggesting that this finding was unlikely to be a function of the particular group of participants. There was a slightly higher proportion of students in Study 1 (63%) and Study 3 (63%), compared with Study 2 (42%), but a one-way ANOVA indicated that occupation was not linked with state anxiety, F (1, 211) = 1.44, *p* = 0.232. Furthermore, as all studies were completed in the same laboratory, with the same conditions (including staff and internal laboratory processes), we must consider alternative explanations for the clinically significant levels of state anxiety prior to bed for every condition in Study 2. As Study 2 was conducted in the latter half of 2016 (whereas Study 1 was performed in the first half of 2016, and Study 3 in the first half of 2017), external global events, seasonal differences or differences in workload (e.g., student examinations) in the latter half of 2016 may have contributed to the significantly higher levels of state anxiety across all conditions in Study 2. However, the reason for this difference in state anxiety between studies is unclear.

When we investigated the effect of trait anxiety on sleep, state anxiety and cognitive outcomes over the different on-call conditions of each study, while there were several statistically significant results, the degree to which any one variable was affected is minimal. Furthermore, it is important to note that the effect of condition was, in each case, found to be small. These findings will be discussed in the context of each study individually.

Study 1 investigated the effects of the likelihood of being called (definitely or maybe) and found that there was no relationship between trait anxiety and either state anxiety or performance outcomes under these conditions. However, in the control condition, there was a non-significant trend for higher levels of trait anxiety to be associated with longer sleep latencies. When this is compared with definitely being called a condition of this study, this relationship is no longer present. However, as this trend was not statistically significant, it is likely that the differences seen under control as compared with on-call conditions were minimal. Additionally, in the definitely condition, lower levels of trait anxiety were associated with a higher number of arousals during REM (both overnight and per hour), when compared with these relationships in the control condition. It must be noted, however, that these differences are likely due to several outliers (i.e., participants who had >50 overnight arousals). Based on these findings, there is a slight trend for individuals with higher trait anxiety to fall asleep faster and to have fewer arousals when they know they will definitely be called—though we must be cautious in our interpretation of these findings. While the trends seen in these data are contrary to the original hypothesis, it is possible that the statistical significance seen is based on spurious associations. These findings may, however, be reflective of the higher baseline level of anxiety that these individuals experience, meaning that when faced with on-call scenarios, they are less reactive than those who have lower levels of baseline anxiety [40]. This is supported by research under stressful conditions, where individuals with higher levels of trait anxiety displayed higher emotional reactivity to a stressful situation, but lower levels of physiological reactivity (cortisol) than individuals with lower trait anxiety [40]. As heightened cortisol appears to be linked with poor sleep outcomes [41], it is possible that under stressful conditions (i.e., knowing that the individual will definitely be called), individuals with lower trait anxiety in fact have higher levels of physiological reactivity, and as such, poorer sleep outcomes than those with higher trait anxiety.

In Study 2, results indicated that under control conditions, there was a non-significant trend of high trait anxiety to be associated with lower state anxiety. This was significantly different from the relationship under the high stress condition. In the high stress condition, there was no apparent relationship between trait and state anxiety. However, while this is a statistically significant trend, the magnitude of the changes in state anxiety is very small between control and the high stress condition (within 5 points), suggesting that this difference is largely inconsequential. There was also a relationship of trait anxiety with time taken to reach N3 sleep in the high stress condition. In the high stress condition, higher trait anxiety was linked with a longer latency to N3 compared with control—however, this finding can only be seen when outliers are included in the dataset. Combined with the latency to N3 differing by less than 10 min between conditions, and the proportion or amount of N3 that was obtained remaining the same, this does not appear to be reflective of poorer sleep overall. Further, there were no significant effects of trait anxiety on any performance outcomes for Study 2. This may also be explained by the lack of cortisol reactivity in higher anxiety individuals under stressful conditions [40]. When we look at the relationship between trait anxiety and state anxiety and sleep outcomes in these studies, it is important to understand the size of these differences in a real-world context. Specifically, the magnitude of the changes in these outcomes is small for all variables and, as such, should be considered trends only. For example, the difference in latency to N3 seen in Study 2 is only representative of an additional sleep latency of under 10 min for individuals with a STAI X-2 score that was 20 points higher.

Though no relationship was found between trait anxiety and either state anxiety or any sleep variables for Study 3, significant differences were found in performance outcomes. In the control condition, individuals with higher trait anxiety had slower reciprocal reaction times (RRT), mean slowest 10% of RRT, and more lapses (though lapse effects were only seen due to outliers), though no relationship between trait anxiety and performance was seen in either on-call condition. Being on-call, regardless of the chance of missing the alarm or quality/quantity of sleep, resulted in improved cognitive performance for individuals with high trait anxiety, but did not change performance for those with lower trait anxiety. These performance changes improved the cognitive performance of higher trait anxiety individuals to the same level as those with lower trait anxiety. One explanation for this change is that individuals who have higher levels of trait anxiety are less physiologically reactive to stressful situations (i.e., lower levels of hormones of the hypothalamo–pituitary–adrenocortical axis, epinephrine, norepinephrine and prolactin) [42]. This may be explained in part by the higher levels of anxiety they experience day-to-day, which may blunt the hormonal response to acute stress. Specifically, under stressful conditions (i.e., attending a motor vehicle collision), individuals with higher trait anxiety actually had lower levels of these physiological stress symptoms, which may be linked with performance [42]. As such, under the stressful on-call conditions, individuals with higher trait anxiety may be more habituated to the physiological responses, and may respond better than those with lower trait anxiety. It is also important to consider the magnitude of these results—a 20-point increase on the STAI X-2 (trait anxiety) resulted in cognitive performance that was impaired by approximately two lapses, and an increase in reaction time of 100 ms. These differences, while significant, are not large in magnitude. Additionally, under certain on-call conditions, this performance advantage may disappear. Further research into the effects of trait anxiety on performance on-call is certainly warranted, as with the additional stressors of real world on-call work, these differences may be more severe.

While this set of interrelated studies was designed to look at the effects of different aspects of on-call work (likelihood of receiving a call, task stress, chance of missing the alarm), the separation of these elements means that this study is limited in terms of real-world applicability. Real on-call workers often experience these factors concurrently, with the addition of actual calls occurring overnight, resulting in sleep restriction. As such, future research must address the effect of combined or additional stressors, in addition to including participants that are more representative of on-call workers as a whole (i.e., women and individuals over the age of 35). It is necessary to include individuals who are experiencing higher levels of depression, stress, or anxiety in future research, as this may be more representative of real world on-call populations. Furthermore, trait anxiety is just one individual factor that may play a role in on-call suitability, and as such, future research must examine other personality and individual factors that may play a role. We must also consider the potential impact of trait anxiety on different cognitive and performance outcomes, other than the simple assessment tools included in the current study (i.e., PVT, polysomnography). Future research should assess the relationship between trait anxiety and complex work performance outcomes such as decision making and teamwork.

This study was the first to investigate the effects of trait anxiety on state anxiety, sleep, and cognitive performance outcomes under a range of on-call conditions. Findings show limited changes to anxiety, sleep, and cognitive performance under on-call conditions. The effects of trait anxiety on state anxiety and sleep outcomes were very small in magnitude and may not have any practical implications for on-call work. However, the trend seen in Study 3 suggests that being on-call may blunt the cognitive performance disadvantage that individuals with higher trait anxiety appear to have under control conditions. As such, having higher, non-clinical levels of trait anxiety does not appear to preclude individuals from being able to cope with on-call work. However, future research is needed to investigate the impact of trait anxiety on complex on-call work performance outside of the laboratory environment.

## Figures and Tables

**Table 1:** Participant demographics.

Characteristic	Study		
	1	2	3
Age (years)	27.7 ± 6.4	27.0 ± 4.1	25.0 ± 3.8
BMI (kg/m^2^)	23.3 ± 1.9	23.7 ± 2.8,	23.6 ± 3.0
PSQI global score	2.5 ± 1.3	2.5 ± 1.2	2.5 ± 1.3
Epworth Sleepiness Scale score	4.0 ± 2.2	4.0 ± 2.0	3.9 ± 2.4
Morningness Eveningness Questionnaire score	54.4 ± 8.0	54.0 ± 7.2	54.8 ± 7.0
Self-reported pre-study bed time (h ± SD (min)) *	2304 ± 53	2245 ± 58	2259 ± 44
Self-reported pre-study wake time (h ± SD (min)) *	0758 ± 60	0750 ± 64	0738 ± 58
Total sleep time in week preceding participation (h) **	7.2 ± 1.1	7.2 ± 1.1	7.0 ± 1.1

* Self-reported over the previous month, based on PSQI item; ** Based on actigraphy corroborated with sleep diaries.

**Table 2:** The relationship between trait anxiety and on-call state anxiety in Study 1, Study 2 and Study 3 compared with control.

Study	Condition	β *	SE	df	t	*p* **	*p* ***
Study 1	Control	0.29	0.29	33.56	1.01	0.322	
	Definitely	0.61	0.23	44.00	1.40		0.169
	Maybe	0.44	0.23	44.00	0.65		0.521
Study 2	Control	−0.37	0.19	31.75	−1.95	0.060	
	High stress	−0.04	0.13	41.39	2.43		0.019
	Low stress	−0.11	0.13	41.39	1.93		0.061
Study 3	Control	0.06	0.23	35.438	0.25	0.801	
	Low chance	0.42	0.19	44.00	1.92		0.061
	High chance	0.35	0.19	44.00	1.58		0.121

* Estimated effect of trait anxiety on state anxiety (interaction term); ** Significance of the relationship between trait anxiety and state anxiety in the control condition (compared with zero); *** Significance of the relationship between trait anxiety and state anxiety on on-call nights compared with control relationship.

**Table 3:** Significant effects of trait anxiety on on-call sleep variables in Study 1, 2, and 3 compared with control.

Variable	Study	Condition	β *	SE	df	t	*p* **	*p* ***
Sleep latency	Study 1	Control	0.67	0.45	46.26	1.50	0.141	
		Definitely	−0.40	0.47	44.00	−2.30		0.026
		Maybe	0.45	0.47	44.00	−0.48		0.635
	Study 2	Control	−0.40	0.44	44.13	−0.90	0.372	
		High stress	−0.07	0.47	40.00	0.69		0.492
		Low stress	0.33	0.47	40.00	1.54		0.131
	Study 3	Control	0.15	0.43	55.81	0.35	0.731	
		Low chance	0.13	0.51	44.0	−0.04		0.966
		High chance	0.71	0.51	44.0	1.09		0.280
Latency to N3	Study 1	Control	0.15	0.25	39.75	0.62	0.541	
		Definitely	0.52	0.23	44.00	1.56		0.126
		Maybe	0.09	0.23	44.00	−0.30		0.764
	Study 2	Control	−0.47	0.52	57.31	−0.90	0.371	
		High stress	1.26	0.68	40.00	2.54		0.015
		Low stress	−0.09	0.68	40.00	0.57		0.574
	Study 3	Control	0.12	0.17	43.05	0.74	0.464	
		Low chance	0.13	0.16	44.0	0.02		0.985
		High chance	0.16	0.16	44.0	0.22		0.825
REM Arousals (overnight)	Study 1	Control	−0.06	0.64	31.03	−0.09	0.927	
		Definitely	−1.15	0.45	44.00	−2.42		0.020
		Maybe	−0.93	0.45	44.00	−1.94		0.058
	Study 2	Control	0.26	0.54	27.26	0.48	0.636	
		High stress	0.75	0.36	40.00	1.35		0.186
		Low stress	0.75	0.36	40.00	1.35		0.185
	Study 3	Control	0.13	0.51	33.85	0.26	0.797	
		Low chance	0.29	0.40	44.00	0.39		0.701
		High chance	−0.44	0.40	44.00	−1.42		0.164
REM Arousals (per hour)	Study 1	Control	0.06	0.31	31.09	0.18	0.860	
		Definitely	−0.47	0.22	44.00	−2.36		0.022
		Maybe	−0.43	0.22	44.00	−2.21		0.032
	Study 2	Control	0.28	0.25	26.61	1.12	0.274	
		High stress	0.46	0.16	40.00	1.14		0.263
		Low stress	0.37	0.16	40.00	0.57		0.571
	Study 3	Control	0.06	0.24	33.42	0.27	0.789	
		Low chance	0.06	0.19	44.00	−0.01		0.993
		High chance	−0.14	0.19	44.00	−1.12		0.267

* Estimated effect of trait anxiety on state anxiety; ** Significance of the relationship between trait anxiety and state anxiety in the control condition (compared with zero); *** Significance of the relationship between trait anxiety and state anxiety on on-call nights compared with control relationship.
